# Role of Absorbable Polysaccharide Hemostatic Powder in the Prevention of Bleeding and Wound Events after Thyroid Surgery

**DOI:** 10.3390/jcm12175684

**Published:** 2023-08-31

**Authors:** Giovanni Docimo, Marcello Filograna Pignatelli, Sonia Ferrandes, Alessandro Monaco, Francesco Calisti, Roberto Ruggiero, Salvatore Tolone, Francesco Saverio Lucido, Luigi Brusciano, Simona Parisi, Giovanni Conzo, Ludovico Docimo, Claudio Gambardella

**Affiliations:** 1 Unit of Thyroid Surgery, Department of Medical and Advanced Surgical Sciences, University of Campania “Luigi Vanvitelli”, 80138 Naples, Italy; giovanni.docimo@unicampania.it (G.D.); marcello.filogranapignatelli@unicampania.it (M.F.P.); sonia.ferrandes@unicampania.it (S.F.); alessandro.monaco@unicampania.it (A.M.); francesco.calisti@unicampania.it (F.C.); 2 Division of General, Oncological, Mini-Invasive and Obesity Surgery, University of Campania “Luigi Vanvitelli”, 80131 Naples, Italy; roberto.ruggiero@unicampania.it (R.R.); salvatore.tolone@unicampania.it (S.T.); francescosaverio.lucido@unicampania.it (F.S.L.); luigi.brusciano@unicampania.it (L.B.); simona.parisi@unicampania.it (S.P.); giovanni.conzo@unicampania.it (G.C.); ludovico.docimo73@gmail.com (L.D.)

**Keywords:** total thyroidectomy, neck seroma, neck haematoma, postoperative bleeding, thyroid drainage

## Abstract

Background: Bleeding is one of the most fearsome and life-threatening complications after thyroid surgery. Several medical devices and haemostatic agents have been proposed to improve haemostasis during total and hemi-thyroidectomy. Resorbable polysaccharide powder (HaemoCer™) is a plant-based polymer that is helpful in terms of the coagulation cascade becoming a gel and forming a barrier to prevent further bleeding, having tested for haemostasis in different districts. The aim of the current study was the evaluation of drain output, the presence of significant postoperative blood loss and complications in patients treated with or without resorbable polysaccharide powder during thyroid surgery. Methods: From January to December 2022, postoperative bleeding, drainage output and the postoperative wound events of patients undergoing thyroid surgery, in a tertiary centre, with haemostasis completion with resorbable polysaccharide powder (Group A) or not (Group B), were retrospectively analysed. Results: Eighty-one patients in Group A received a haemostasis improvement with the use of reabsorbable polysaccharide powder, and 96 patients in Group B received thyroid surgery alone. Patients in Group A presented lower drainage output (0.005), lower incidence of neck haematoma (0.005) and seroma (0.021), confirmed also by multivariate analysis. Conclusions: The resorbable polysaccharide powder, in the current series, appeared to be an effective agent in achieving haemostasis in thyroidectomies, reducing the postoperative drainage output, and also neck events such as neck haematoma and seroma, improving the postoperative comfort of the patients. Further larger comparative studies are needed to address this issue.

## 1. Introduction

Haemostasis is a physiological process triggered by blood extravasation, which involves various procoagulant and fibrinolytic factors. The intrinsic pathway, activated through exposed endothelial collagen, and the extrinsic pathway, activated through tissue factor released by endothelial cells after external damage, are well-known processes able to clot blood [1]. Bleeding is one of the terrible complications of thyroid surgery, with an incidence of 0.1–3.8% [2]. Several factors contribute to the risk of bleeding in thyroid surgery. These factors include the size and location of the thyroid gland, the presence of thyroid nodules or goitre, the skill and experience of the surgeon and the patient’s overall health and coagulation status. During the surgical procedure, the surgeon takes measures to minimize bleeding and achieve haemostasis. This typically involves the careful dissection and identification of blood vessels, the ligation or cauterization of vessels to seal them off and the use of haemostatic techniques and agents as needed. Another frequent sequela after thyroid surgery is neck seroma. It results from an accumulation of serous fluid under the skin flaps. The pathogenesis of seroma formation is due to the accumulation of acute inflammatory exudate in response to surgical trauma during a prolonged acute phase of healing. Therefore, new medical devices or agents able to assure haemostasis have been largely advocated. In addition to the classic bleeding prevention methods, such as compression, mono or bipolar electric scalpel, clips, ligatures and modern dissection and synthesis devices, natural and synthetic topical haemostatic agents have been studied, produced and used for several years, and are appreciated for their feasibility of use and efficacy. These have been divided into haemostats and surgical sealants, and differ in terms of their materials, composition, methods of preparation, use and conservation.

Among these haemostatics, resorbable polysaccharide powder (HaemoCer™, BioCer Entwicklungs-GmbH, Bayreuth, Germany) is a plant-based polymer derived using purified vegetable starch, which is helpful in starting the coagulation cascade by absorbing the blood serum and increasing the concentration of the platelets and coagulation factors, and with the formation of a gel adhering and forming a barrier to prevent further bleeding. The safety and efficacy of this haemostat powder can be used in areas difficult to reach or blocked in various surgery [3,4,5,6,7], obtaining consensus in the scientific community. 

The aim of the current study is the evaluation of drain output after 48 h, the presence of significant postoperative blood loss and complications in patients treated with or without resorbable polysaccharide powder during thyroid surgery.

## 2. Methods

### 2.1. Study Design

This study was reported according to the STROBE statement for cohort studies [8] and it was conducted according to the ethical principles stated in the Declaration of Helsinki. Written informed consent was obtained from all patients. 

### 2.2. Study Setting and Study Population

Patients referred to the Unit of Thyroid Surgery of the University of Campania “Luigi Vanvitelli”, from 1 January 2022 to 31 December 2022, for a diagnosed thyroid disease and who were undergoing total thyroidectomy (TT) or hemithyroidectomy (HT) were considered in the current study. Inclusion criteria included: thyroid benign or malignant disease; American Society of Anesthesiologists (ASA) physical status of grade ≤ IV [9]

Exclusion criteria included: pregnancy, previous thyroid cancer, distant metastases; autoimmune thyroid disease, previous tumours of the head-neck district, neck surgery within 5 years, neck radiation therapy, chronic inflammation due to HBV, HCV, chronic gastritis, nephritis or gout, immune or haematological disease, proliferative haemopoietic disorders, heart failure and the use of an anticoagulant drug. These conditions could represent a bias to verify the effectiveness of the haemostat. 

All patients underwent a preoperative blood sample, a neck ultrasound, a fine needle cytology (FNC) of the thyroid neoplasm and fibreoptic-laryngoscopy tests in the 30 days prior to surgery. After referral for surgery, each patient received a detailed explanation of the procedure by the medical staff and had to sign a personalised informed consent form. All the operations were performed by the same experienced surgeons.

Clinical data were collected in an electronic database and retrospectively analysed. Patients were divided into two groups: Group A, patients with the application of the product and aspiration drainage, and Group B, patients with only aspiration drainage, which was considered the control group. During the procedure, the application of the haemostat was decided by the local condition and whether there was a necessity for advanced haemostasis. Particularly, the case of diffuse or extensive bleeding made it challenging to achieve haemostasis through conventional methods, such as sutures or electrocautery alone, especially in dangerous areas (i.e., Zuckerkandl tubercle) where the risk of intraoperative nervous or parathyroid lesion is higher. Moreover, it was applied in case of recurrent or persistent bleeding during thyroid surgery, despite the surgeon’s best efforts.

Clinical and demographical parameters (age, sex, BMI, thyroid volume, type of intervention, intraoperative blood loss, volume of drainage and thyroid disease) were evaluated, as well as the postoperative outcomes (day of discharge, rates of complications, onset of seroma or haemorrhage, reoperation). The follow-up consisted of regular clinical and instrumental examinations of the neck until the twenty-eighth day following discharge.

### 2.3. Surgical Technique

The conventional sutureless TT or HT technique was always followed by a suction drain positioning; no additional procedures were performed in the control group (Group B) [10,11,12]. In Group A, 5 g of Haemocer^TM^ was administered for bleeding that did not respond to surgical haemostasis, in particular in the Gruber and Sappey ligaments, avoiding electrocautery injuries to recurrent nerves. Furthermore, in Group A patients, a suction surgical drain was placed. All patients received the same postoperative management. The surgical drain was removed after 48 h. 

### 2.4. Outcome Measures

The mean operative time was reported in minutes and median hospitalization was reported in days. The mean drainage output was evaluated in millilitres. Postoperative hypoparathyroidism was defined as the presence of postoperative serum calcium < 8 mg/dl via blood test. Postoperative vocal fold palsy was clinically suspected in cases of shortness of breathing and vocal modification, and was assessed via laryngoscopy. The presence of any other postoperative complications (i.e., seroma or hematoma formation, wound infection) was assessed at the outpatient clinics, and was expressed as the number of cases and as a percentage. Neck ultrasonography was performed in case of clinical reservations regarding seroma or blood collection. 

### 2.5. Study Endpoints

The primary endpoint was the drain output (mL) after 48 h and the presence of significant blood loss (if the patient needed to return to the operating room). Secondary endpoints included the evaluation of the presence of seroma, hematoma, the duration of surgery and the postsurgical complications.

### 2.6. Statistical Analysis

Continuous variables were described as mean and standard deviation, while categorical variables were described as the number of cases. The population was divided into two groups, whether or not a haemostat had been used. Independent sample *t*-tests were performed to compare the continuous variables (age, days of discharge, BMI, thyroid volume in mL, operation time and drainage volume); a Test of Proportions was applied to the categorical variable of seroma formation. Moreover, Fisher’s exact test was used in order to analyse gender and malignant or benign disease. The Odds Ratio (OR) was calculated using univariate and multivariate Logistic Regression. Statistical significance was considered in the case of a *p*-value < 0.05. Statistical analysis was performed with SPSS version 23 (SPSS©, Chicago, IL, USA).

## 3. Results

### 3.1. Population

From 1 January 2022 to 31 December 2022, 219 patients received thyroid surgery for thyroid pathology. One hundred and seventy-seven met the inclusion criteria and were considered in the study. Patients were divided into two groups, according to the use of the haemostat; 81 in Group A, where haemostasis was completed with the use of reabsorbable polysaccharide powder, and 96 in Group B, without the use of any haemostatic agent. Demographic and clinical data are detailed in Table 1. 

The mean age was 49.44 ± 15.34 in Group A and 51.13 ± 15.26 in Group B, showing no statistical difference. In Group A, 67 patients received TT, while 14 patients underwent HT. In Group B, 71 patients were treated with TT and 25 patients with HT [Table 2]. The mean operation time was statistically different (68.4 ± 37.64 in Group A and 75.88 ± 14.78 in Group B, *p* = 0.031). The mean hospital stay was 3.12 ± 0.55 in Group A and 3.24 ± 0.96 in Group B (*p* = 0.079). At definitive pathology, a malignant disease resulted in 32 patients in Group A (39.5%) and in 35 patients in Group B (36.4%) (*p* = 0.677).

### 3.2. Primary Outcomes

The drain output within 48 h was 65.9 ± 38.58 mL vs. 92.13 ± 51.91 mL in Groups A and B, respectively, with a statistically significant difference (*p* = 0.005). Postoperative bleeding requiring surgical revision occurred in one patient undergoing TT (1.2%) of Group A and four patients of Group B, without reaching statistical significance.

### 3.3. Secondary Outcomes

Regarding secondary outcomes, eight patients (9.8%, one after HT and seven after TT) presented neck seroma in Group A vs. twenty-two (22.9%, three after HT and nineteen after TT) patients in Group B (*p* = 0.021); a neck haematoma, not requiring a surgical revision, occurred in two patients (2.4%, all TT) in Group A and fourteen patients (14.6%, two HT and twelve TT) in Group B (*p* = 0.005). It was conservatively managed with simple aspiration under US during the outpatient visit. Moreover, the operative time resulted in 68.4 ± 37.64 min in Group A and 60.88 ± 14.78 min in Group B (*p* = 0.357). No wound infection, transient or permanent bilateral or unilateral Vocal Cord palsy or hypoparathyroidism were recorded. No mortality was reported in either group.

Univariate and multivariate analyses of drainage volume and seroma formation were evaluated, showing odds ratio (OR) of 0.988 and 0.591 (*p*-value < 0.05) for univariate analysis and 0.977 and 0.579 (*p*-value < 0.05) for multivariate analysis. The day of discharge showed no statistical difference in terms of OR [Table 3].

## 4. Discussion

Thyroid surgery is one of the most frequent surgical procedures worldwide, but it has several complications, such as recurrent laryngeal nerve palsy, superior laryngeal nerve injury and parathyroid insufficiency. Bleeding is one of the most dangerous and life-threatening sequelae, with an incidence, according to the literature, ranging from 0% to 4% [2,10,11]. In the case of massive bleeding, an emergent intervention is required, since post-operative hematoma could obstruct the airway due to larynx compression and impairment through venous and lymphatic drainage [13,14]. Haemorrhage during thyroid surgery could occur in three specific moments: intraoperative haemorrhage; haemorrhage within 24 h from the beginning of the procedure; postoperative haemorrhage delayed 7–10 days after surgery due to the erosion of a vessel [13,15]. Seroma formation is another complication, due to the accumulation of acute inflammatory exudate in response to surgical trauma during prolonged healing [16,17].

Conventional techniques for haemostasis are: suture and/or ligatures, electrocautery or surgical clips and, of course, compression. Today, the most modern dissection and synthesis devices could permit a sutureless thyroidectomy [10,11,12], but in order to ensure haemostasis and to avoid injuries to anatomical structures, such as the recurrent laryngeal nerve and parathyroid, haemostatic agents have been widely used [17,18]. Haemostatic agents can be classified according to their origin (animal, human, plants and synthetic derivatives) and they can be used as topical haemostats, which clot a bleeding surface with either thrombin or fibrinogen, separately used or in combination, or as sealants, preventing leakage, or as adhesives [19]. The choice of haemostatic agent depends on factors such as the surgeon’s preference, the severity of the bleeding, the location of the bleeding and the patient’s overall condition. Surgeons may use a combination of different agents to achieve optimal haemostasis during thyroid surgery. It is important to note that the use of haemostatic agents should be guided by the surgeon’s expertise and based on individual patient factors. By employing appropriate haemostatic agents and techniques, surgeons can effectively control bleeding during thyroid surgery, minimize postoperative complications and contribute to successful patient outcomes. Resorbable polysaccharide powder (HaemoCer™ Plus) is a plant-based polymer derived from purified vegetable starch; it is a topical haemostat, very feasible and quick to use; the blood serum is absorbed by the powder and increases the concentration of the platelets and coagulation factors, developing a gel, which adheres and forms a barrier to prevent further bleeding [3]. During thyroid surgery, HaemoCer™ can be directly applied to the bleeding site or used to pack areas with difficult haemostasis. It adheres to the tissue, creating a physical barrier that promotes clot formation and provides temporary haemostasis. As a resorbable powder, it eventually dissolves within the body and is absorbed, leaving no residue or foreign material behind. However, it is important to note that the choice and application of haemostats should be judiciously carried out, considering the individual patient’s condition, the extent of surgery and the surgeon’s expertise. Close attention must be paid to potential adverse reactions or complications associated with the specific haemostatic agent used.

To the best of our knowledge, the current study is the first and largest study to analyse the postoperative complications in thyroid surgery after the use of reabsorbable polysaccharide powder. In the current series, in fact, despite the fact that haemostatic powder was adopted in cases of bleeding not responding to surgical haemostasis, a massive haemorrhage requiring a surgical revision only occurred in one case in Group A (1.2%) and in four cases in Group B (3.1%); however, the difference was not statistically different (*p* = 0.240). Possibly, apart from the effectiveness of the powder, the intraoperative bleeding occurrence determined subsequent careful and meticulous research of the haemorrhage sources, limiting the postoperative onset of the complication. Neck seroma occurred in eight cases in Group A (9.8%) and twenty-two patients in Group B (22.9%), showing a statistically significant difference (*p* = 0.021). Moreover, a relevant statistical difference (*p*-value = 0.005) was observed between the groups regarding the drainage volume (65.9 ± 38.58 vs. 92.13 ± 51.91 in Groups A and B, respectively). Furthermore, operation time showed a statistical difference between the two groups: 68.4 ± 37.64 vs. 75.88 ± 14.78 in Groups A and B, respectively (*p* = 0.031). Possibly, the adoption of a haemostatic agent to improve complete haemostasis helped to achieve an easier and faster method. 

Univariate and multivariate analyses were performed, showing a concrete OR of drainage volume (univariate analysis: 0.988 C.I. 95% 0.979–0.997; *p*-value = 0.007; multivariate analysis: 0.977 C.I. 95% 0.972–0.999 *p*-value = 0.005). The seroma formation OR had a statistical significance (univariate analysis: 0.591 C.I. 95% 0.380–0.919; *p*-value = 0.018; multivariate analysis: 0.579 C.I. 95% 0.412–0.985; *p*-value = 0.015). The discharge day OR did not show a statistical difference in either univariate or multivariate analysis. These data confirmed that the haemostatic agent could be a protective factor and reduce the drainage output and the probability of seroma complications during follow-up after thyroid surgery. 

In the literature, the safety and efficacy of microporous polysaccharide in reducing the seroma rate was demonstrated in murine models [20]. In other studies, no correlation has been found between the use of this haemostatic agent, drainage output and seroma complications [15,16], even in thyroid surgery [3], but the populations were limited compared to the current study. In other surgery fields, evidence of reducing the complication rate has been observed [6]. Larger studies are needed to determine whether using a haemostatic agent such as HaemoCer™ could be useful to prevent post-operative hematomas, even in procedures carried out by less experienced surgeons; however, the results of our study might provide a valuable basis by which to evaluate the incidence of complications such as hematomas, seromas and infection [21,22].

Moreover, HaemoCer could be reabsorbed very quickly, without leaving any remnants and reducing the probability of false positives of thyroid tissue persistence during the follow-up, different from the use of a fibrillar resorbable haemostatic agent. 

This study has some limitations to address, such as the retrospective design of the study and the limited number of analysed patients. Moreover, being a retrospective study, we reported the surgical experience analysing the clinical records of our surgical division in patients that received the haemostat powder or not. Of course, this could present a bias related to the intraoperative conditions of the patients. Therefore, the conclusions are hardly generalizable. 

## 5. Conclusions

Postoperative bleeding after thyroid surgery is a fearsome and life-threatening complication and devices and products able to limit it are still being investigated. Resorbable polysaccharide powder, in the current study, appeared to be an effective agent in achieving haemostasis in thyroidectomies, and reducing postoperative neck haematoma and bleeding. Moreover, it decreased the drain output and seroma formation during follow-up, improving the postoperative comfort of the patients. Further and larger comparative studies are needed to address this issue.

## Figures and Tables

**Table 1:** The baseline clinical and demographic features in both groups. BMI, Body Mass Index; ASA, American Society of Anesthesiologists (ASA).

	Group A 81 Patients	Group B 96 Patients	*p*-Value
Age	49.44 ± 15.34	51.13 ± 15.26	0.273
Gender (Male/Female)	22/59 (27.2%/72.8%)	34/62 (35.4%/64.6%)	0.239
BMI	26.91 ± 4.85	27.84 ± 3.31	0.263
ASA I-II/III	69/12 (85.2%/14.8%)	81/15 (84.3%/15.7%)	0.881
Hypertension	22 (27.1%)	31 (32.3%)	0.430
Diabetes	11 (13.5%)	15 (15.6%)	0.701
Chronic obstructive pulmonary disease	3 (3.7%)	7 (7.2%)	0.303
Cerebrovascular Disease	2 (2.4%)	3 (3.1%)	0.793
Heart Ischemic Attack	4 (4.9%)	7 (7.2%)	0.518
Smoking	21 (25.9%)	34 (35.4%)	0.174
Anticoagulant/antiplatelet drugs	18 (22.2%)	25 (26.1%)	0.555

**Table 2:** Intraoperative and Postoperative Outcomes in Group A and B. TT, total thyroidectomy; HT, hemithyroidectomy.

	Group A 81 Patients	Group B 96 Patients	*p*-Value
Surgical Procedure	67 TT (82.7%) 14 HT (17.3%)	71 TT (73.9%) 25 HT (26.1%)	0.161
Operation Time (min)	68.4 ± 37.64	75.88 ± 14.78	0.031
Thyroid volume (mL)	29.97 ± 7.45	32.01 ± 3.45	0.119
Definitve Pathology Malignant Disease Benign Disease	32 (39.5%) 49 (60.5%)	35 (36.4%) 61 (63.6%)	0.677
Drainage volume (mL)	65.9 ± 38.58	92.13 ± 51.91	0.005
Reoperation	1 (1.2%)	4 (3.1%)	0.240
Day of discharge	3.12 ± 0.55	3.24 ± 0.96	0.079
Neck Seroma	8 (9.8%)	22 (22.9%)	0.021
Neck Haematoma	2 (2.4%)	14 (14.6%)	0.005
Mortality	0	0	-

**Table 3:** Univariate and multivariate analyses evaluating the odds that haemostat reduces drainage output and day of discharge. ^a^ Adjusted for age, sex, BMI, procedure, operation time and thyroid volume. (OR, odds ratio; CI, confidential interval). * Statistically Significant.

	Unadjusted OR (95% CI)		Adjusted OR ^a^ (95% CI)	
		*p*-Value		*p*-Value
Drainage volume	0.988 (0.979–0.997)	0.007 *	0.977 (0.972–0.999)	0.005 *
Days of discharge	1.705 (0.905–3.213)	0.099	2.115 (0.942–5.176)	0.061
Seroma formation	0.591 (0.380–0.919)	0.018 *	0.579 (0.412–0.985)	0.015 *

## Data Availability

The datasets used and/or analysed during the current study are available from the corresponding author on reasonable request.
